# CsCDPK6, a CsSAMS1-Interacting Protein, Affects Polyamine/Ethylene Biosynthesis in Cucumber and Enhances Salt Tolerance by Overexpression in Tobacco

**DOI:** 10.3390/ijms222011133

**Published:** 2021-10-15

**Authors:** Heyuan Zhu, Meiwen He, Mohammad Shah Jahan, Jianqiang Wu, Qinsheng Gu, Sheng Shu, Jin Sun, Shirong Guo

**Affiliations:** 1 College of Horticulture, Nanjing Agricultural University, Nanjing 210095, China; zhuheyuan1030@163.com (H.Z.); shahjahansau@gmail.com (M.S.J.); 2017204044@njau.edu.cn (J.W.); shusheng@njau.edu.cn (S.S.); jinsun@njau.edu.cn (J.S.); 2 Institute of China Agricultural University Press, China Agricultural University, Beijing 100094, China; meiwen0408@163.com; 3 Zhengzhou Fruit Research Institute, Chinese Academy of Agricultural Sciences, Zhengzhou 450009, China; guqsh@126.com

**Keywords:** cucumber, salt stress, calcium-dependent protein kinase, S-adenosylmethionine synthetase (SAMS), PA/ethylene metabolism

## Abstract

S-adenosylmethionine synthetase (SAMS) plays a crucial role in regulating stress responses. In a recent study, we found that overexpression of the cucumber gene *CsSAMS1* in tobacco can affect the production of polyamines and ethylene, as well as enhancing the salt stress tolerance of tobacco, but the exact underlying mechanisms are elusive. The calcium-dependent protein kinase (CDPK) family is ubiquitous in plants and performs different biological functions in plant development and response to abiotic stress. We used a yeast two-hybrid system to detect whether the protein CDPK6 could interact with SAMS1 and verified their interaction by bimolecular fluorescence complementation (BiFC) and co-immunoprecipitation (Co-IP) assays. To further explore the function of cucumber *CDPK6*, we isolated and characterized *CsCDPK6* in cucumber. CsCDPK6 is a membrane protein that is highly expressed under various abiotic stresses, including salt stress. It was also observed that ectopic overexpression of *CsCDPK6* in tobacco enhanced salt tolerance. Under salt stress, *CsCDPK6*-overexpressing lines enhanced the survival rate and reduced stomatal apertures in comparison to wild-type (WT) lines, as well as lowering malondialdehyde (MDA) and hydrogen peroxide (H_2_O_2_) contents and causing less relative electrolyte leakage. Moreover, repression of *CsCDPK6* expression by virus-induced gene silencing (VIGS) in cucumber seedling cotyledons under salt stress increased ethylene production and promoted the transformation from putrescine (Put) to spermidine (Spd) and spermine (Spm). These findings shed light on the interaction of *CsSAMS1* and *CsCDPK6*, which functions positively to regulate salt stress in plants.

## 1. Introduction

Cucumber is one of the most important vegetable crops widely grown in the world. However, cucumber root distribution is relatively shallow, fragile and sensitive, and its tolerance to salt stress is poor. Therefore, the yield and quality of cucumber are often reduced due to salt damage during cultivation. Plant exposure to salt stress increases the contents of some protective substances and expressions of some salt stress-related genes and proteins, which improve the salinity resistance of plants.

Polyamines (PAs) are low-molecular weight aliphatic nitrogen-containing alkaloids with strong biological activity, which are produced during the metabolic process of organisms and are widely distributed in plant tissues. When plants are subjected to abiotic stresses, the contents of PAs change rapidly, and different types and forms of PAs can be transformed into one another in response to stresses. PAs biosynthesis and signaling are involved in the regulation of antioxidant enzymes, H_2_O_2_ levels, ion channels and balancing ion homeostasis [1]. Currently, PAs are used as plant growth regulators to increase plant salt stress tolerance [2,3]. For ethylene, there are few studies on its synthesis pathway in plant responses to stress, and the specific regulatory mechanism is not clear. Studies have found that a double mutant of *ein3-1 eil1-1*, a downstream transcription factor of EIN2 in the ethylene signaling pathway, is salt-sensitive under salt stress in Arabidopsis thaliana [4]. S-adenosylmethionine (SAM) is the precursor for the synthesis of PAs in plant cells. The synthesis of *SAM* is solely catalyzed by S-adenosylmethionine synthase (SAMS) from methionine and ATP [5,6]. A series of studies have shown that *SAMS* is involved in plant biotic and abiotic stress responses. For example, the interaction between the stress signaling molecule ABA, H_2_O_2_ and NO mediates the expression of *SAMS* under low temperature stress [7], and the expression of *SAMS* is upregulated in plants during resistance to infection of dodders [8]. Salt stress can significantly inhibit or induce the expression of *SAMS* in cucumber, suggesting that *SAMS* is involved in the regulation of cucumber salt stress tolerance [9,10]. There have been few studies performed on the interaction of SAMS proteins in plants to date. Rice F-box protein OsFBK12 interacts with OsSAMS1, and OsSAMS1 is degraded as a target substrate by OsFBK12 through the 26S proteasome pathway, affecting the ethylene level in the plant and regulating leaf senescence and grain size [11]. A plasma membrane receptor-like protein kinase, Fer, in *Arabidopsis* interacts with AtSAMS1 and AtSAMS2 to negatively regulate ethylene synthesis [12]. Similarly, two plasma membrane receptor-like protein kinases, MdFERL1 and MdFER6, in apple regulate the changes in ethylene content during the fruit development and ripening processes by interacting with MdSAMS [13]. AtCPK28 can interact with AtMAT1, AtMAT2 and AtMAT3 (AtSAMS) and degrade AtMAT1, AtMAT2 and AtMAT3 through the 26S proteasome pathway, regulating ethylene synthesis and lignin deposition in *Arabidopsis thaliana* [14]. SAMS catalyzes the synthesis of PAs and ethylene, and it is also involved in the synthesis of nicotinamide, lignin, betaine and other metabolites [5,15,16].

Calcium-dependent protein kinases (CDPKs or CPKs) are ubiquitous in plants and involved in a variety of calcium-mediated signaling pathways through auto-phosphorylation and targeted protein phosphorylation [17,18,19,20]. They also act on the downstream of proteins involved in plant stress responses. *AtCPK10* participates in stomatal regulation of *Arabidopsis thaliana* under drought conditions [21]. In rice, ABA induces *OsCPK4* expression, and *OsDi19-4* acts as a downstream response gene of *OsCPK14* that positively regulates the expression of ABA response genes in rice [22]. In addition, *CDPKs* also play a key role in stress-induced oxidative damage in *Arabidopsis* by participating in reactive oxygen degradation. The *cpk1*, *cpk4*, *cpk5* and *cpk6* mutants reduce ROS production under stress. *CDPKs* activate NADPH oxidase through phosphorylation and play a key role in stress-induced oxidative damage. In rice, *OsCPK12* regulates ROS homeostasis under salt stress by inducing the expression of the ROS-scavenging gene *OsAPX2*/*OsAPX8* and inhibiting the NADPH oxidase gene *OsRBOHI* [23].

*CDPKs*, as a gene family with important functions in plant signal transduction pathways, have become a research hotspot in cucumber and other plants. Exploring the mechanisms of *CDPK* family genes when regulating plant signaling pathways and participating in stress responses can provide a more adequate theoretical basis for revealing various life activities in the plant life cycle and also lay a theoretical foundation for molecular breeding. Cucumber *CDPK* family genes are involved in many processes of plant growth and development and are expressed in different tissues at different growth and development stages. When plants are faced with biological or abiotic stresses, *CDPK* family gene-mediated signaling pathways respond to these stimuli, transmitting signals or participating in regulation, such as salt stress, drought, high temperature and low temperature stress and ABA. It has been proved that *CDPKs* are an important gene family involved in the regulation of plant life activities. The study of *CDPK* genes can provide high-quality gene resources for the cultivation of stress-resistant cucumber varieties. There are also some difficult problems to overcome in the study of the function of *CDPK* family genes, and due to the large number of family genes and functional redundancy, the interaction network of related proteins is relatively complex. Our previous research isolated and characterized the *CsSAMS1* gene from cucumber and explored its response to a variety of abiotic stresses [24]. To further explore the relationship between *CsSAMS* and *CsCDPK* and the potential role of *CDPKs* in salt stress tolerance, a *CDPK* gene in cucumber was investigated in this study, namely, *CsCDPK6* [25]. Therefore, this study will further explore the function of cucumber *CDPKs* in regulating plant responses to salt stress, including the following two aspects: verifying the interaction between cucumber SAMS1 and CDPK6 proteins; exploring the expression pattern of *CDPK6* in cucumber, and studying the response and alleviating mechanism of *CDPK6* in cucumber under salt stress by measuring relevant physiological indicators, expression of salt-tolerant genes and metabolism levels of polyamines and ethylene. Thus, this study’s purpose is to reveal the regulation function of the *CsCDPK6* gene on the polyamine ethylene metabolism pathway in cucumber under stress, and the interaction regulation mechanism between CDPK6 and SAMS1 in cucumber.

## 2. Results

### 2.1. SAMS1 Interacts with CDPK6

To explore the function of *SAMS1* in cucumber, a yeast two-hybrid (Y2H) screening assay was conducted for candidate CsSAMS1-interacting proteins. A cucumber root library was constructed under salt stress conditions for this study. The full length of *CsSAMS1* was fused into pGBKT7 as a bait, in order to screen a cucumber cDNA library. Several independent positive proteins were isolated, and a CDPK was identified as a potential CsSAMS1-interacting protein. The full length of *CsCDPK6* was cloned by PCR with primers designed using a database for cucumber.

**Figure 1:**
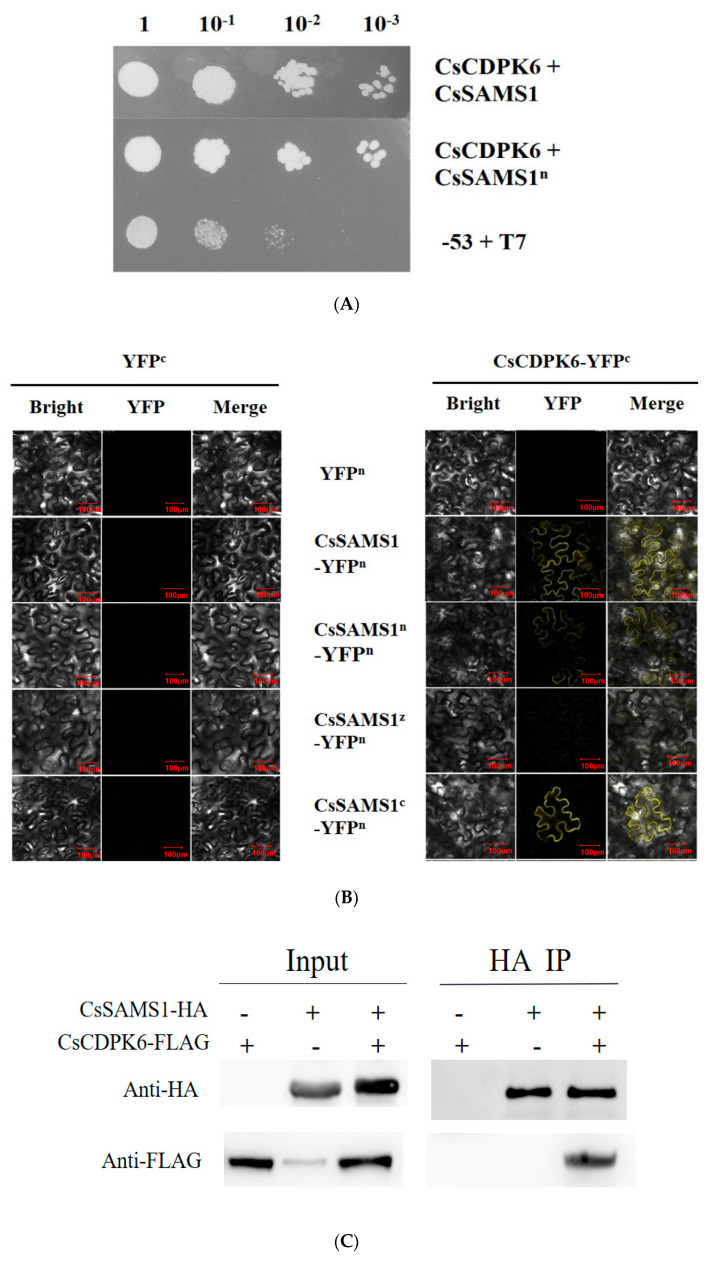
Interaction between CsCDPK6 and CsSAMS1. (**A**) Y2H assays showing the interaction between CsCDPK6 and CsSAMS1. (**B**) BiFC assay confirming the interaction between CsCDPK6 and CsSAMS1 in vivo. YFP field indicates fluorescence signals. Scale bar = 100 μm. (**C**) Co-immunoprecipitation assay. Immunoblot analysis confirmed the expression of input proteins: CsSAMS1-HA (panel 1, anti-HA antibody), CsCDPK6-Flag (panel 2, anti-Flag antibody); the IP assay was incubated with anti-HA-tag magnetic beads, and CsCDPK6-Flag was detected only after co-immunoprecipitation with the sample expressing CsSAMS1-HA (panel 3, anti-FLAG antibody).

Additionally, we also performed a Y2H screening test. AD-CsCDPK6 and BD-CsSAMS1 were co-transformed into Y2H Gold yeast, and the yeast grew on an SD/-Leu/-Trp/-His/Ade plate. The N-terminal, C-terminal and intermediate domains of CsSAMS1 were constructed into the pGBKT7 vector and co-transfected with AD-CsCDPK6 into Y2H Gold yeast. The interaction between CsSAMS1 and CsCDPK6 was detected in CsSAMS1 (Figure 1A). To confirm the result of the interaction between CsSAMS1 and CsCDPK6 in vivo, a bimolecular fluorescence complementation (BiFC) assay and co-immunoprecipitation (Co-IP) test were conducted. CsSAMS1 and its N-terminal, C-terminal and intermediate domains were constructed into the N-terminal of YFP, and CsCDPK6 into the C-terminal of YFP. The results show that CsSAMS1 and its N-terminal, C-terminal and intermediate domains could interact with CsCDPK6. From the brightness of the YFP signal, it could be preliminarily concluded that an interaction existed between the N-terminals of CsSAMS1 and CsCDPK6 (Figure 1B).

To support these observations, we used a co-immunoprecipitation (Co-IP) assay to verify their interaction. Immunoblot (IB) analyses with an anti-HA antibody revealed the interaction between CsCDPK6-Flag and CsSAMS1-HA in vivo (Figure 1C).

### 2.2. Phylogenetic and Expression Analysis of CsCDPK6

We obtained the full-length cDNA of *CsCDPK6* (GenBank accession no. MH922008) from cucumber (*Cucumis sativus* L.) line ‘9930’ by performing RT-PCR. Sequence analyses showed that the *CsCDPK6* gene’s CDS length is 1629 bp, of which the ORF is 984 bp in length and encodes a protein of 543 amino acids. It has a predicted molecular weight of 61.51 kDa and an isoelectric point of 8.75. Analysis of the secondary structure of *CsCDPK6* with the SMART tool revealed a Serine/Threonine protein kinase catalytic domain (91–351 aa), and two EF hand calcium binding motifs (398–426 aa, 477–505 aa). *CsCDPK6* was located on chromosome 6 and contained 12 exons.

**Figure 2:**
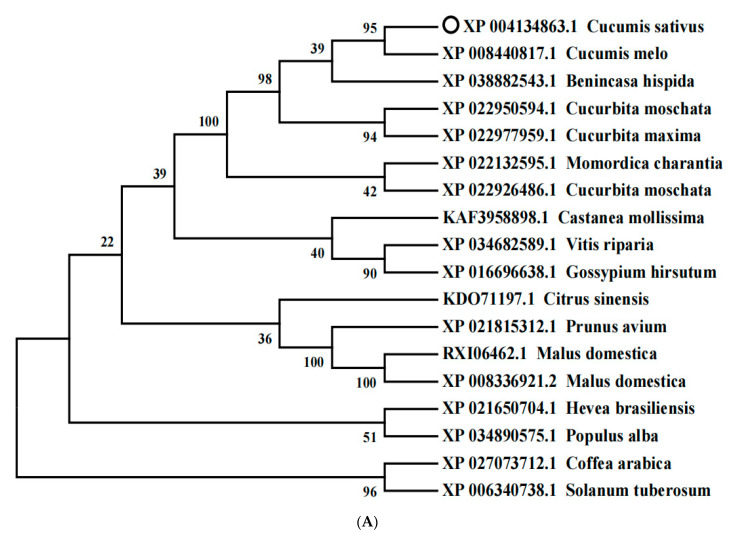

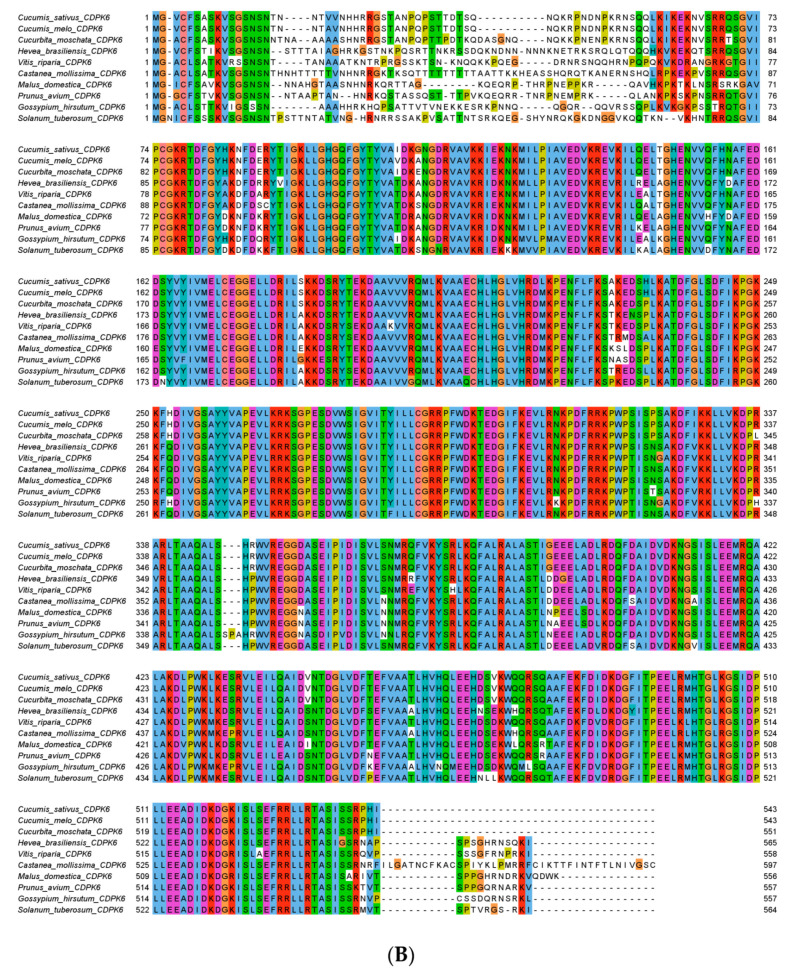
Phylogenetic analysis of CsCDPK6. (**A**) Phylogenetic analysis of CsCDPK6 and related CDPK6 homologs in different species. (**B**) Sequence alignment of CDPKs among different species.

To better understand the evolutionary relationships between *CsCDPK6* and *CDPKs* from other plants, a phylogenetic tree was constructed using the amino acid sequences of *CDPKs* from 18 species via MEGA 7.0 software (Figure 2A). *CsCDPK6* and *CmCDPK4* (XP 008440817.1) were found to have the closest evolutionary relationship. Multiple alignments of the predicted residues of CDPK proteins showed that CsCDPK6 proteins displayed a similarity to other species of CDPK, where the C-terminal and middle amino acid sequence consistency was 86.88%; however, the N-terminal sequence consistency was 16.9% (Figure 2B), indicating that those CDPKs had similar functions but different subcellular localizations.

**Figure 3:**
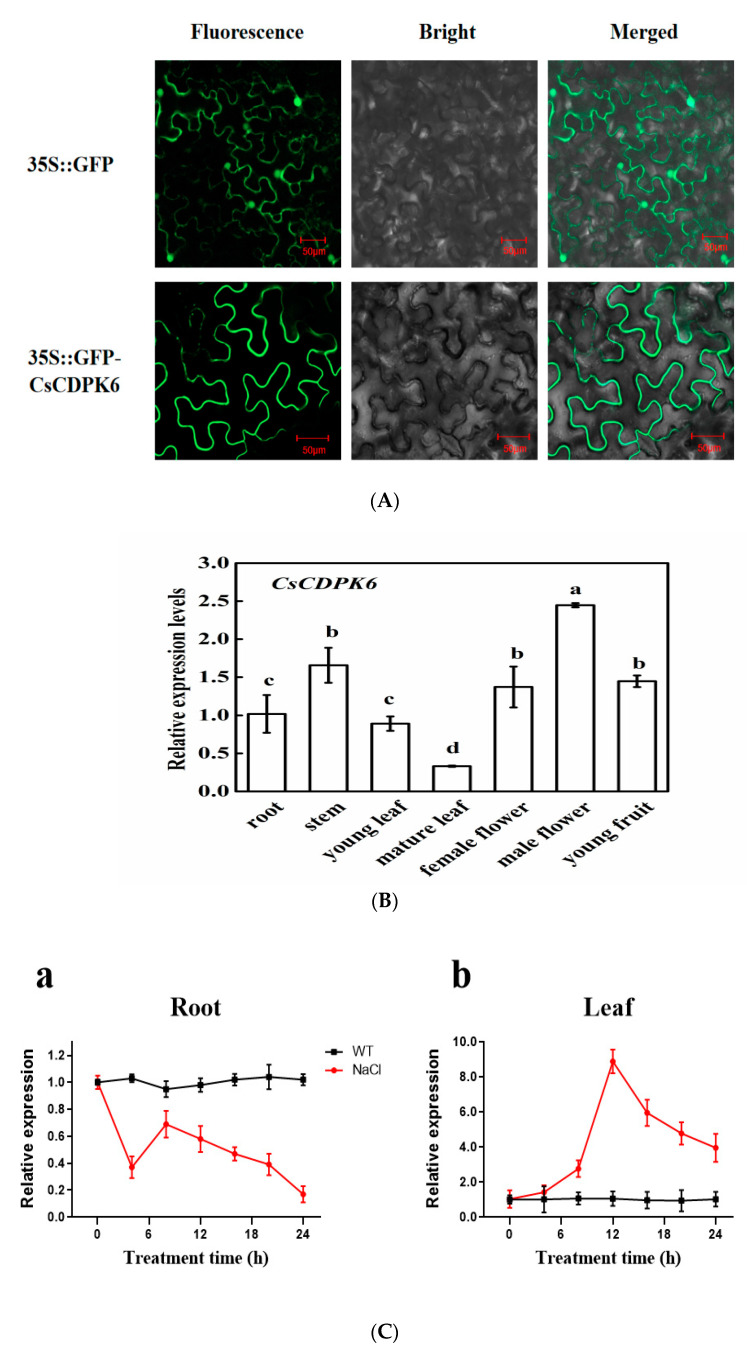
(**A**) Subcellular localization of *CsCDPK6*. Scale bar = 50 μm. (**B**) Expression patterns of cucumber *CsCDPK6* in various organs. (**C**) Expression patterns of *CsCDPK6* over 24 h in roots and leaves under salt stress. Different letters indicate expression levels that were significantly different at *p* < 0.05.

To explore the localization of *CsCDPK6* in cells, the *CsCDPK6* recombinant vector with a GFP tag was injected into leaves of tobacco (*N. benthamiana*). In the cell of transgenic leaves, the GFP fluorescence of *CsCDPK6* was located in the plasma membrane (Figure 3A).

In order to further analyze the expression patterns of *CsCDPK6*, we selected different tissues and organs of cucumber using quantitative real-time PCR (qPCR). As shown in Figure 3B, the expression level of *CsCDPK6* was the highest in male flowers, followed by female flowers and young fruits of stems, and the lowest was in mature leaves. This result suggests its possible function in reproductive organs. To know whether *CsCDPK6* plays a role in salt stress, roots and leaves were exposed to salt stress conditions. Salt stress reduced the expression of *CsCDPK6* in roots and enhanced the expression of *CsCDPK6* in leaves (Figure 3C). Under salt stress, the expression level of *CsCDPK6* in cucumber roots was more stable than that in leaves. The expression level of *CsCDPK6* in leaves reached a peak at 6 h but was still higher than the control plants at 24 h.

**Figure 4:**
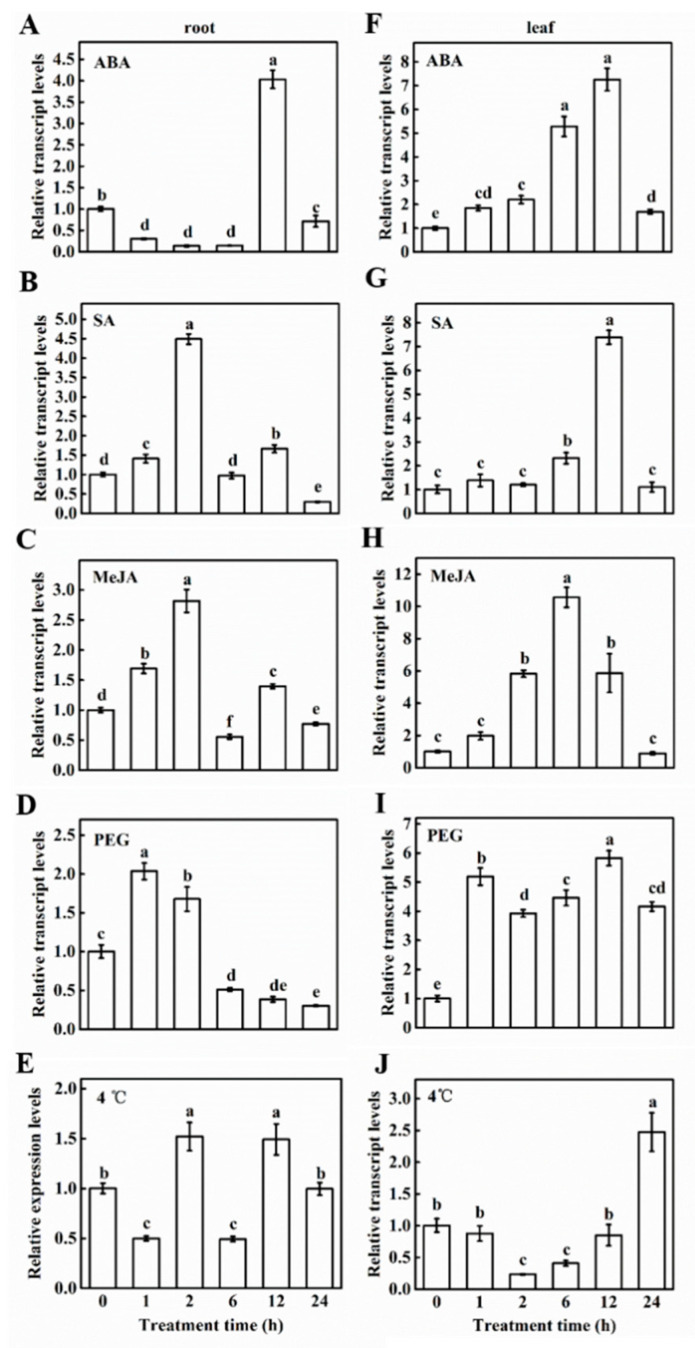
CsCDPK6 expression level in cucumber roots and leaves under different hormones and abiotic stresses. (**A**,**F**) 100 μM ABA; (**B**,**G**) 100 μM SA; (**C**,**H**) 100 μM MeJA; (**D**,**I**) 20% PEG (*w*/*v*); (**E**,**J**) 4 °C. Different letters indicate expression levels that were significantly different at *p* < 0.05.

In addition, we further found that its expression levels changed in response to hormones and abiotic stresses, specifically for ABA, SA, MeJA, PEG and cold treatments. Exogenous ABA treatment rapidly induced the expression of *CsCDPK6* in cucumber seedling leaves, and the expression level was 5.2- and 7.2-fold higher than the control at 6 h and 12 h, respectively. The effect in roots was firstly downregulated and then dramatically increased, and the induced amount at 12 h was four times the control level (Figure 4A,F). Exogenous SA treatment enhanced the expression of *CsCDPK6* in roots and leaves of cucumber seedlings, being 4.5- and 7.2-fold higher than the control at 2 h and 12 h, respectively (Figure 4C,B,G). The effect of exogenous MeJA treatment on *CsCDPK6* expression was similar to SA treatment, and the peak of *CsCDPK6* appeared earlier in roots than in leaves (Figure 4C,H). The expression of *CsCDPK6* in roots did not rise for a long time after 20% PEG treatment, and the expression of *CsCDPK6* was lower than the control after 6 h of treatment. In contrast, 20% PEG treatment rapidly and continuously induced the expression of *CsCDPK6* in leaves, and the expression level within 24 h was significantly higher than that in the control leaves (Figure 4D,I). The expression of *CsCDPK6* was not significantly affected by low temperature treatment in roots of cucumber seedlings (Figure 4E,J).

### 2.3. Overexpression of CsCDPK6 Enhances Tobacco Tolerance to Salt Stress

**Figure 5:**
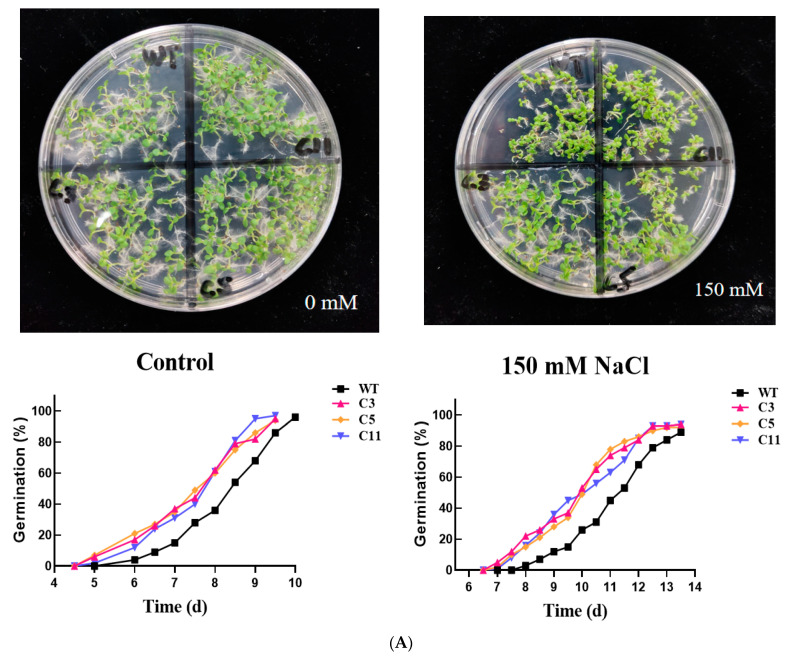

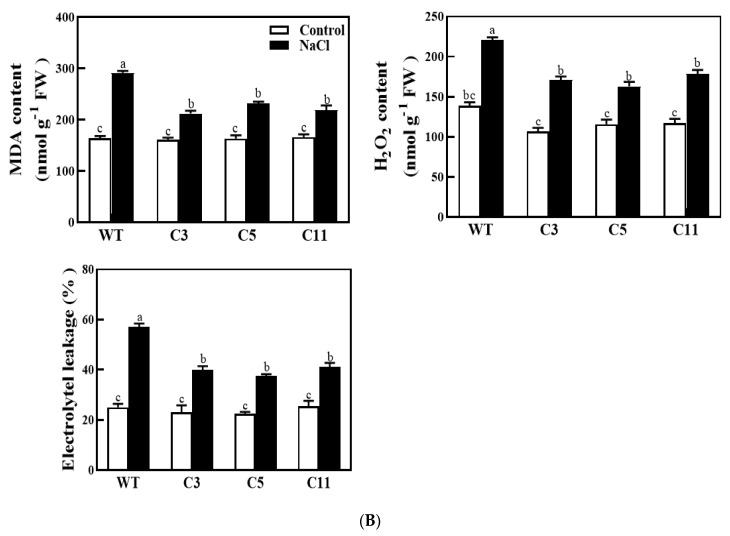
Overexpression of *CsCDPK6* enhanced salt stress tolerance in tobacco. (**A**) Seed germination rate of wild-type and *CsCDPK6* transgenic plants. (**B**) MDA content, H_2_O_2_ concentration and electrolyte leakage in *CsCDPK6*-overexpressing lines and WT plants under salt stress. Different letters indicate expression levels that were significantly different at *p* < 0.05.

To further explore the role of *CsCDPK6* under stress, transgenic tobacco plants overexpressing *CsCDPK6* were generated. We used uniformly transgenic lines C3, C5 and C11 as experimental material. First, we compared the seed germination of WT and *CsCDPK6*OE plants after NaCl treatment (Figure 5A). The germination rate of *CsCDPK6*OE seeds decreased sharply after 150 mM NaCl treatment but was still significantly higher than WT. Then, membrane lipid peroxidation and antioxidant analysis were conducted on WT and *CsCDPK6*OE tobacco seedlings treated with salt stress for 3 d. The degree of membrane lipid peroxidation could be reflected by the content of malondialdehyde (MDA) and H_2_O_2_. As shown in Figure 5B, there was no significant difference in MDA and H_2_O_2_ contents between *CsCDPK6*OE and WT observed under control conditions. After 150 mM NaCl treatment, MDA and H_2_O_2_ contents in WT tobacco leaves were significantly higher than those in *CsCDPK6*OE transgenic lines by 32.7% and 27.2%, respectively, indicating that *CsCDPK6*OE tobacco lines under salt stress had lower membrane lipid peroxidation production, which reflected the degree of the membrane system and cell damage. The electrolyte leakage of WT leaves was significantly higher than that of transgenic tobacco after salt stress treatment, indicating that cell damage of WT plants was more serious than that of transgenic lines.

**Figure 6:**
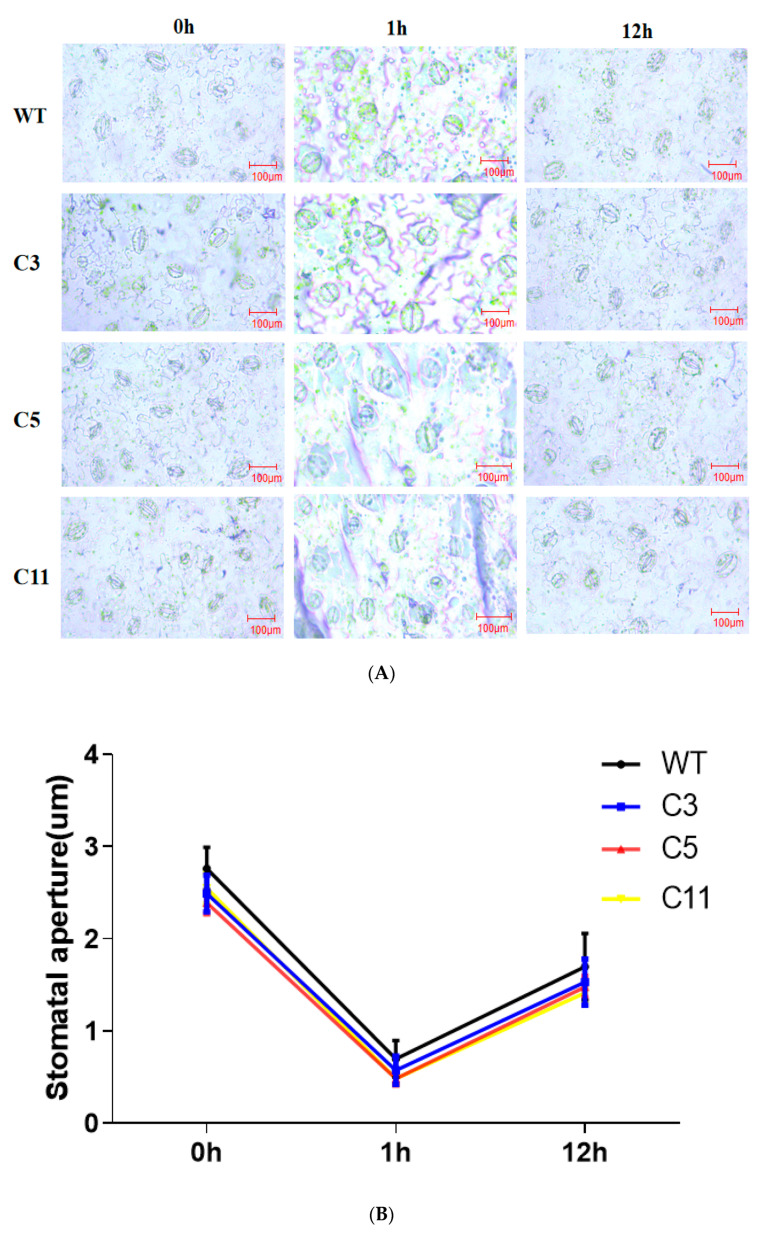
Determination of stomatal aperture under salt stress. (**A**) Stomatal aperture of the lower epidermis of leaves of wild-type and *CsCDPK6*-overexpressing lines after salt treatment under living electron microscopy interference line after being under salt stress. (**B**) Stomatal aperture of leaf lower epidermis of wild-type and *CsCDPK6*-overexpressing lines.

The leaf stomatal density and opening degree of wild-type and *CsCDPK6*-overexpressing lines were measured and statistically compared under an electron microscope. The results showed that the average stomatal density of WT lines was 70.88/mm^2^, while that for the *CsCDPK6*OE lines was 52.78/mm^2^, which is significantly lower than that for the WT lines. The results of stomatal opening showed (Figure 6A) that before salt treatment, the average stomatal opening of wild-type WT lines was 2.94 μm, and that for *CsCDPK6*OE lines was 2.47 μm. After 1 h of salt treatment, most stomata were closed, and the average stomatal opening of WT was 0.65 μm, and that for transgenic lines was 0.5 μm. After 12 h of salt treatment, some stomata were reopened, and the measurement results show that the average stomatal opening of WT was 1.86 μm, while that for transgenic lines was 1.56 μm. The average stomatal opening of *CsCDPK6*OE lines was significantly lower than that of WT lines (Figure 6B). In conclusion, *CsCDPK6*OE can improve salt stress resistance of tobacco by changing the stomatal density and opening.

### 2.4. Silencing of CsCDPK6 Regulates PA and Ethylene Metabolism in Cucumber

**Figure 7:**
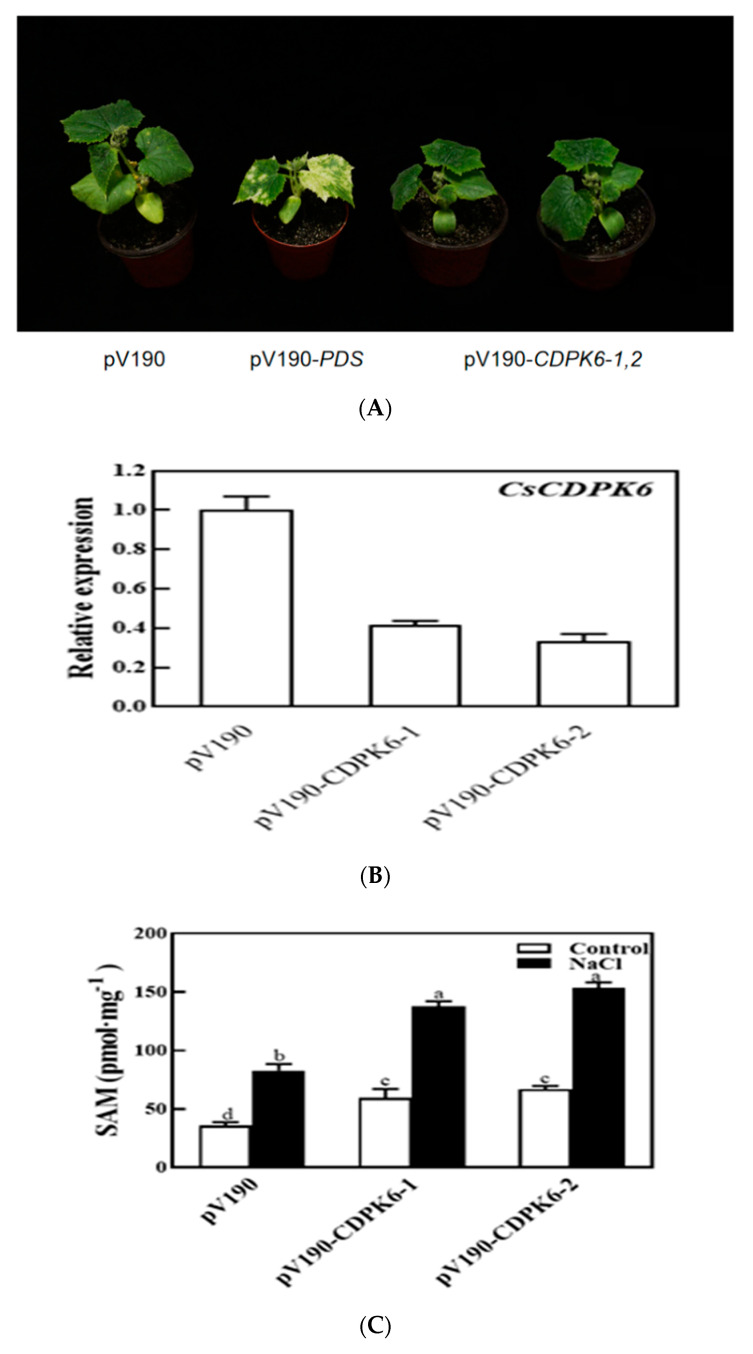

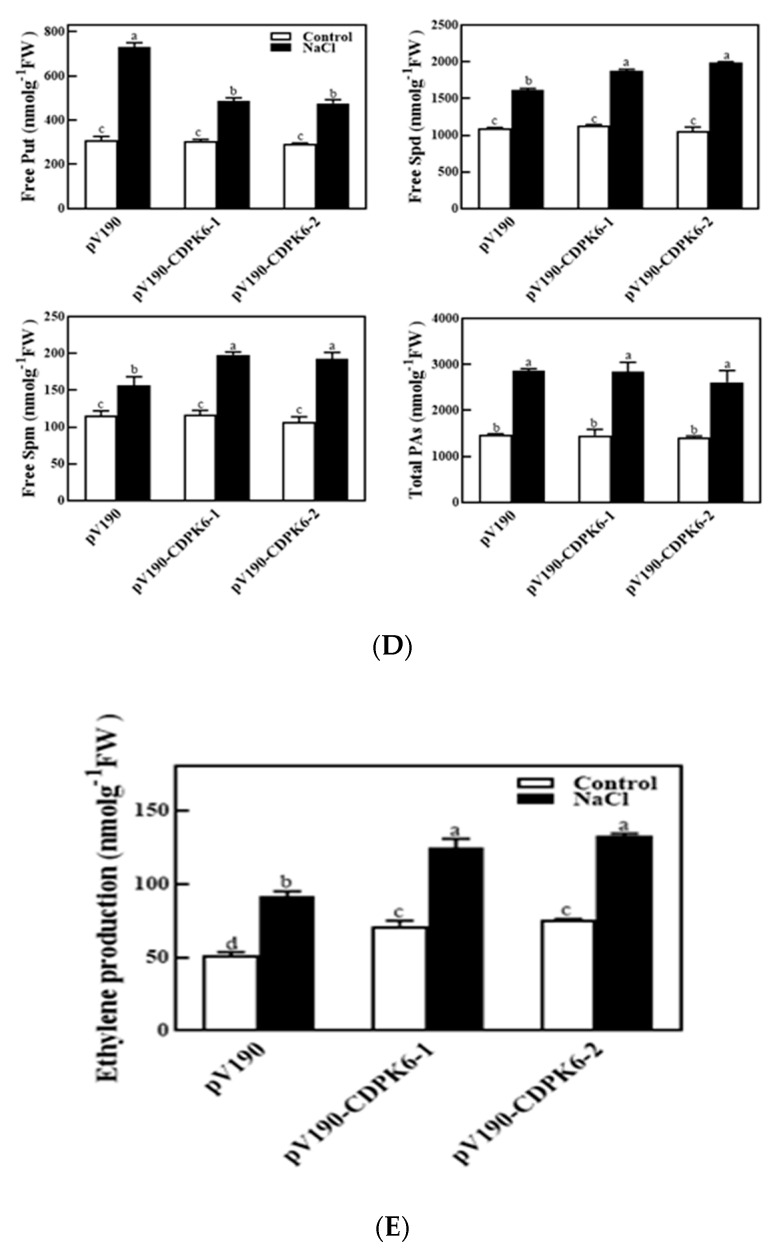
Silencing of *CsCDPK6* regulated PA and ethylene metabolism in cucumber. (**A**) Phenotype of VIGS lines. (**B**) Relative expression level of *CsCDPK6* in VIGS plants. (**C**) SAM content in *CsCDPK6*-silenced lines under salt stress. (**D**) Free PA content in *CsCDPK6*-silenced lines under salt stress. (**E**) Ethylene content in *CsCDPK6*-silenced lines under salt stress. Different letters indicate expression levels that were significantly different at *p* < 0.05.

To gain insight into the further functions of *CsCDPK6*, we generated *CsCDPK6*-silenced cucumber lines by virus-induced gene silencing (VIGS). A 300 bp fragment of *CsCDPK6* was inserted into the pV190 vector, which was then transformed into Agrobacterium. After 30 d, the leaves of pV190-*PDS* lines showed photobleanching. *CsCDPK6* was significantly reduced at transcript levels by 60–75% compared to pV190 empty vector lines (Figure 7A,B), suggesting *CsCDPK6* was efficiently silenced for use in further research.

SAM is the precursor of PA and ethylene synthesis in plants. The contents of SAM changes in WT and *CsCDPK6*-silenced plants were measured to explore the role of *CsCDPK6* under salt stress. Under control conditions, the content of SAM in *CsCDPK6-*silenced plants was significantly higher than that of WT. After salt stress treatment, the SAM content in *CsCDPK6*-silenced lines was significantly higher than that in WT plants (Figure 7C), indicating that *CsCDPK6* negatively regulated endogenous SAM synthesis.

Salt stress can induce endogenous PAs in plants [26,27,28]. In order to explore whether *CsCDPK6* is involved in the regulation of endogenous PA production under salt stress, the content of PA changes was further analyzed. Under control conditions, the contents of free Put, Spd and Spm in *CsCDPK6*-silenced lines showed no significant difference compared with WT. Under salt stress, the contents of Put in *CsCDPK6*-silenced lines were decreased, while the contents of Spd and spermine in *CsCDPK6*-silenced lines were increased significantly, which were higher than those in WT. The total amount of free PAs showed no significant difference compared with WT, indicating salt stress could promote the transformation from Put to Spd and spermine in *CsCDPK6*-silenced lines (Figure 7D).

For ethylene metabolism, there was no difference in the ethylene content in *CsCDPK6*-silenced lines compared with WT under normal conditions. After 150 mM NaCl treatment, ethylene metabolism in *CsCDPK6*-silenced lines was induced, which was significantly higher than that in WT plants (Figure 7E), indicating that the inhibiting the expression of the *CsCDPK6* gene could increase the endogenous ethylene content of cucumber.

## 3. Discussion

SAMS is a unique enzyme catalyzing the synthesis of SAM, which is not only an important methyl donor but also a common precursor for the synthesis of PAs and ethylene. In this report, we demonstrated the interaction between CsSAMS1 and CsCDPK6 by Y2H and BiFC techniques. It was also found that the N-terminal and C-terminal domains of CsSAMS1 contributed to its interaction with CsCDPK6 to varying degrees, and the interaction signal between the N-terminal and CsCDPK6 was the strongest. According to the principle of Y2H technology, when two proteins with a weak interaction are close to each other in space, it may not be enough to activate transcription and trigger simultaneous expression of multiple reporter genes, as shown in Figure 1A. The N-terminal domain of fused CsSAMS1 and Y2H Gold yeast of CsCDPK6 grew well on the defect plate, which is consistent with the growth of Y2H Gold yeast of the fused CsSAMS1 complete sequence and CsCDPK6, suggesting that the N-terminal domain of CsSAMS1 is important for its interaction with CsCDPK6. In this study, when the interaction signals were detected under confocal microscopy, it was found that the experimental group expressing the N-terminal domain had the strongest YFP fluorescence signal (Figure 1B). SAMS is a catalytic enzyme containing multiple phosphorylation sites, and it may be phosphorylated as a substrate for kinases; protein kinases typically carry this out by acting on one or more Ser/Thr sites [29,30,31]. Therefore, it needs to be verified whether the interaction between CsCDPK6 and CsSAMS1 is a correlation between kinases and phosphorylated substrates. If so, further exploration is needed to find the specific site of interaction between CsCDPK6 and CsSAMS1.

CDPKs are calcium sensors that can transmit extracellular Ca^2+^ signals to intracellular compartments. CDPKs can interact with target proteins to phosphorylate, causing Ca^2+^ signal cascade amplification and transduction, and participate in plant stress responses to biotic and abiotic stress [32]. Previous studies on cucumber *CDPK* gene families found that different *CsCDPK* expression patterns were different in cucumber organs and tissues, and there were also great differences in response patterns to ABA and various types of stresses [25], indicating that *CsCDPKs* are widely distributed in various tissues and actively participates in the regulation of growth and development and the response to stress. In this study, q-PCR was used to analyze the relative expression level of *CsCDPK6* in male flowers. It is also highly expressed in female flowers, suggesting that it may play an important regulatory role in flower development. *CsCDPK6* in cucumber seedling leaves and roots positively responds to salt, drought, low temperature and various hormone treatments. The results of this study, together with previous studies, suggest that *CsCDPK6* is both constituent and inducible, which may play an important role in plant growth and development and stress responses. In order to explore the function of *CsCDPK6* in plant responses to salt stress, transgenic tobacco *CsCDPK6*OE materials were constructed in this study. Compared with wild-type tobacco plants, the germination rate of *CsCDPK6*OE lines was significantly higher than WT lines under salt stress, while the contents of H_2_O_2_, MDA and EL were significantly lower than WT tobacco plants, which reduced the damage of the cell membrane. This is consistent with *AtCPK1* and *AtCPK6* overexpressed in *Arabidopsis thaliana* that enhancing salt tolerance by regulating ROS and proline production [33,34]. AtCPK13 kinases phosphorylate AtKAT1 and AtKAT2 proteins in *Arabidopsis thaliana*, inhibiting the K^+^ inflow process, closing the guard stoma and reducing leaf water loss [35]. In this study, *CsCDPK6*OE lines improved tobaccos tolerance to salt stress by changing the stomatal density and stomatal opening after salt stress treatment, but whether this process is regulated by Na^+^ and K^+^ flow pathways need to be further proved.

**Figure 8:**
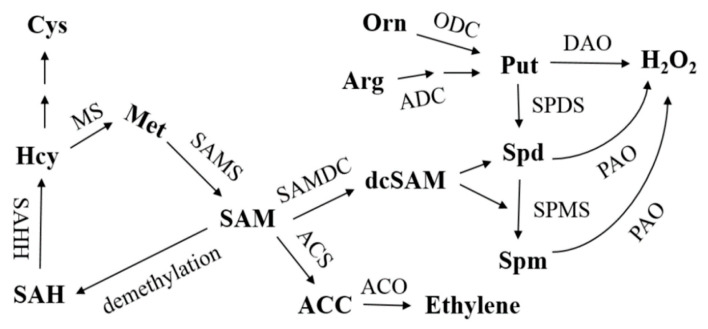
Polyamine and ethylene anabolism pathways in plants.

SAMS is the only catalytic enzyme for SAM biosynthesis. SAM generates aminocyclopropane carboxylic acid (ACC) through 1-aminocyclopropane carboxylic acid synthase (ACS) and then synthesizes ethylene through 1-aminocyclopropane carboxylic acid oxidase (ACO) (Figure 8). In addition, aminopropyl was obtained by SAM decarboxylic acid, which promoted Put in PAs to synthesize Spd and Spm under the catalysis of SPDS and SPMS enzymes. In this experiment, silencing of *CDPK6* promoted ethylene synthesis gene expression and induced endogenous ethylene content to increase in cucumber. Salt stress can induce the production of SAM (Figure 7C) and increase the transformation of endogenous Put to Spd and Spm in cucumber (Figure 7D). From the experimental results, we speculate that the interacting protein CDPK6 could negatively regulate ethylene metabolism in plants by binding to SAMS1 and inhibit the decarboxylation of SAM, thus reducing the transformation of Put to Spd and Spm in PAs. PA metabolism plays a key role in enhancing the stress resistance of cucumber under stress. It can eliminate excessive oxygen free radicals by stabilizing osmotic regulation and regulating the activity of antioxidant enzymes (SOD CAT APX), partially replace SOD to directly eliminate free radicals and alleviate some ion deficits to enhance the salt resistance of cucumber [10,36]. Currently, reported studies on SAMS interaction only focused on the analysis of changes in ethylene synthesis caused by protein kinase phosphorylation of SAMS and did not detect changes in PAs metabolism [37]. This may be because the increase or decrease in ethylene synthesis has a greater effect on the plant phenotype, or because polyamines and ethylene are simply not competitive in inhibition, and plants may also be affected by growth- and stress-related factors in SAM allocation.

SAH: S-adenosine homocysteine; SAHH: S-adenosine homocysteine hydrolase; Hcy: homocysteine; MS: methionine synthase; Met: methionine; SAM: S-adenosine methionine; SAMS: S-adenosine methionine synthase; SAMDC: S-adenosylmethionine decarboxylase; dcSAM: decarboxylation S-adenosine methionine; ACS: aminocyclopropane carboxylic acid synthase; ACC: 1-aminocyclopropane-1-carboxylic acid; ACO: amino cyclopropane carboxylic acid oxidase; ADC: arginine decarboxylase; ODC: ornithine decarboxylase; Put: putrescine; SPDS: spermidine synthase; Spd: spermidine; SPMS: spermine synthase; Spm: spermine; DAO: diamine oxidase; PAO: polyamine oxidase.

## 4. Materials and Methods

### 4.1. Plant Materials and Growth Conditions

Cucumber (*Cucumis sativus* L. cv. Jinchun No. 4) seeds were purchased from Tian Jin Kernel Cucumber Research Institute, Tianjin, China. Seeds were kept for germination on 3 × 3 × 4 cm sponges in the dark for 24 h at 28 °C ± 1 °C. The germinated seeds were then sown in plastic trays (41 × 41 × 5 cm) covered with a mixture of peat and vermiculite (2:1, *v*:*v*). When the first true leaves were fully expanded, the seedlings were transplanted into smart climatic chambers (AEtrium 3; AEssense Corp., Sunnyvale, USA). The growth conditions were maintained as follows: 14 h/10 h (light/dark) photoperiod, 25 °C/18 °C (day/night), 600 µmol m^−2.^s^−1^ photosynthetic photon flux density and 75–80% relative humidity. At the three-leaf stage, plants were treated as follows: 75 mM NaCl as salt stress, 20% polyethylene glycol (PEG) 6000 as drought stress, 4 °C as cold stress, spraying leaves with 100 μM salicylic acid (SA), 100 μM methyl jasmonate (MeJA) and 100 μM abscisic acid (ABA) as hormone treatment. The samples were collected at 0, 1, 2, 6, 12 and 24 h. All the samples were frozen immediately in liquid nitrogen and stored at −80 °C until use for RNA extraction.

### 4.2. Total RNA Extraction and Gene Expression Analysis

Total RNA was isolated from cucumber leaf tissue using RNA simple Total RNA Kit (Tiangen, Beijing, China, DP419) according to the manufacturer’s instructions. Total RNA (1 μg) was used to reverse transcribe cDNA using the HiScript II Q RT SuperMix for qPCRA Kit (Vazyme, Nanjing, China, R223-01). The quantitative real-time PCR (qPCR) assays were performed in the StepOnePlus™ Real-Time PCR System (Applied Biosystems, Foster City, CA, USA).) using the ChamQSYBR qPCR Master Mix (Vazyme, Q311-02, Nanjing, China). The qPCR program contained an initial denaturation at 94 °C for 10 min, followed by 40 cycles at 94 °C for 30 s, 58 °C for 40 s and 72 °C for 30 s. The cucumber *actin* gene was used as an internal control. Gene-specific primers were designed according to cDNA sequences, as described in Table S1. Relative expression was calculated using the 2^−ΔΔCt^ formula [38].

### 4.3. Y2H Screening and Assays

The CDS of *CsSAMS1* was recombined into the bait vector pGBKT7. Y2H screening of the cucumber cDNA library was performed using a yeast transformation system (Clontech, Mountain View, USA,) as described previously [39]. Following the identification of interacting protein *CsCDPK6*, a validation Y2H assay was carried out [39]. The CDSs of *CsSAMS1* and *CsCDPK6* were cloned into the pGBKT7 and pGADT7 vectors, respectively. The primers used in this investigation are listed in Table S1.

### 4.4. BiFC Assays

For BiFC assays analyses, the N-terminal domain (1–108 aa), intermediate domain (109–239 aa), C-terminal domain (240–393 aa) and the CDS sequence of *CsSAMS1* were designed to amplify cucumber SAMS1 protein sequences and fused to the N-terminal of the ZYN vector, and the coding region of *CsCDPK6* was cloned into the ZYC vector. All constructs were transformed into the *Agrobacterium* strain EHA105. Equal volumes of *Agrobacterium* harboring CsCDPK6-YFP^c^ and CsSAMS1-YFP^n^ (or CsSAMS1^n^-YFP^n^, CsSAMS1^z^-YFP^n^, CsSAMS1^c^-YFP^n^) and P19 were mixed to a final concentration of OD_600_ = 0.8. *Agrobacterium* lines were infiltrated in leaves of *Nicotiana benthamiana*. Plants were grown at 23 °C and allowed to recover for 3 d; then, the fluorescence of YFP in the leaves was imaged using a confocal laser scanning microscope (LSM 780, Zeiss, Germany).

### 4.5. Co-IP Assays

The *N. benthamiana* leaves were ground in liquid nitrogen and homogenized in extraction buffer (50 mM Tris-HCl, pH 8.0, 150 mM NaCl, 1 mM EDTA, 0.2% Triton X-100 (Solarbio, T8200), 1 mM phenylmethylsulphonyl fluoride (Solarbio, P8340, Beijing, China), 10 mM dithiothreitol). Extractions were clarified by centrifugation at 12,000 rpm for 30 min at 4 °C, and the protein concentration of the supernatant was adjusted to 1.5 mg/mL. For each precipitation, 500 μg of protein extract was incubated with 200 uL anti-HA magnetic bead suspension (Clontech, Mountain View, CA, USA, Cat. No.635696) for 4 h at 4 °C in a top-to-end rotator. After incubation, the beads were washed four times with ice-cold washing buffer (Clontech, Mountain View, CA, USA, Cat. No.635696) and then eluted by boiling in 2 × SDS loading buffer. Samples were separated by SDS-PAGE and analyzed by immunoblotting with anti-FLAG or anti-HA antibodies.

### 4.6. Determination of Plant Germination Rate

The tobacco seeds were sown on MS medium with or without 150 mM NaCl and then placed in the incubator for 5 d. The germination of the seeds was measured every 24 h, and then the greenness was observed for 14 d. Experiments were repeated three times.

### 4.7. Electrolyte Leakage, Malondialdehyde (MDA) and Hydrogen Peroxide (H_2_O_2_) Determination

The determination method of the electrolyte leakage rate was based on Dhindsa et al. [40]. An amount of 0.5 g fresh leaves was thoroughly washed with deionized water, cut into small pieces, put into tubes filled with 20 mL deionized water and placed at room temperature for 4–5 h under dark conditions in a shaker; then, the initial electrical leakage (EL) in the bathing solution was determined by a portable conductivity meter (DDS-307, Shanghai Precision and Scientific Instrument LTD., Shanghai, China). Subsequently, the samples were boiled at 95 °C for 20 min and cooled to room temperature, and the final electrical conductivity (EC_2_) was measured in the bathing solution. Simultaneously, we determined the deionized water conductivity (EC_0_). The EL was calculated as follows: EL% = (EC_1_ − EC_0_/EC_2_ − EC_0_) × 100.

An amount of 0.2 g fresh leaves was added to 1.6 mL precooled 0.1% TCA, ground in an ice bath to a homogenate and then transferred to a 2 mL centrifuge tube and centrifuged at 12,000× *g* at 4 °C for 20 min. The supernatant obtained was the crude enzyme solution determined by malonaldehyde (MDA) and hydrogen peroxide (H_2_O_2_). MDA content was measured following the method in [41]. Approximately 1 mL crude enzyme solution was taken, and 1 mL 10% TCA (*m*/*v*) containing 0.67% TBA (*m*/*v*) was added into the solution. The mixed solution was boiled in a water bath for 15 min, immediately kept on ice and cooled and then centrifugated at 3000× *g* for 15 min. The light absorption values at 532 nm and 600 nm were measured. MDA content was expressed as nmol/g FW. The H_2_O_2_ content determination was performed according to Alexieva et al. [42]. In short, 500 μL extracted crude enzyme solution was taken, 1 mL 1 M KI and 500 μL PBS-K (100 mM, pH 7.8) were added and then the reaction mixture was kept in the dark for 1 h at room temperature. After that, the mixture solution was centrifuged, an aliquot was used to measure the H_2_O_2_ content and the absorbance was recorded at 390 nm in darkness. The content of hydrogen peroxide in the sample was calculated according to the standard curve of the known concentration of hydrogen peroxide. H_2_O_2_ content was expressed as μmol/g FW.

### 4.8. Measurement of Stomatal Movement

The transgenic and wild-type plants were studied under a microscope to know the stomatal movement after being exposed to salt stress for 1 h and 12 h. The third fully expanded leaf was taken from the top of the tested plant, and a small leaf with an area of about 0.4 cm^2^ was cut from the same part of the leaf with a blade. The stomata of leaf epidermal cells were observed under a light microscope and photographed. We observed 10 visual fields for each group of plants, and 3 replicates were observed for each strain. The stomatal opening in each field was measured using ImageJ software, with the maximum distance between the abdominal walls of guard cells as the stomatal opening value.

### 4.9. SAM Content Determination

The plant samples were ground into powder in liquid nitrogen and extracted with 0.5 mL 0.1 M HCl. Centrifugation at 16,400× *g* at 4 °C was performed twice for 10 min each time to remove cell debris and the cell membrane. The supernatant was washed with chloroacetaldehyde at 80 °C for 10 min: the total volume was 1 mL, in which there was 0.15 mL of sample, and chloroacetaldehyde (3.6%, *v*/*v*) and citrate/phosphate buffer were 0.48 M and 0.59 M, pH 4.0, respectively. The resulting samples were immediately cooled to 4 °C and centrifuged at 16,900× *g* for 1 h. A supernatant of 10 μL was added to the sample, and the content of SAM was detected by an Agilent HPLC system. The excitation and emission wavelengths were set at 280 nm and 410 nm, respectively.

### 4.10. Determination of Free PAs

The contents of PAs in leaves were determined by high-performance liquid chromatography (HPLC) [43]. Approximately 0.5 g sample was taken and ground in an ice bath with 5% (*m*/*v*) perchloric acid ice followed by centrifugation at 12,000× *g* at 4 °C for 20 min. The supernatant was collected to determine the content of free PAs. Around 700 μL of sample was taken into ampoules, 5 mL 6 M HCl was added and the sample was sealed by an alcohol blowtorch. After hydrolysis at 110 °C for 18 h and evaporation at 70 °C, 5% perchloric acid was added to dissolve it again. The ethyl ether phase was extracted with ether, and the ethyl ether phase was collected. After drying, 64% (*v*/*v*) methanol was added to obtain the PA solution. The contents of PAs were detected by an Agilent HPLC system, and the detection wavelength was set at 230 nm.

### 4.11. Determination of Ethylene Content

After weighing plant leaves, the leaves were placed on water-soaked filter paper, and the filter paper was rolled up. The rolled leaves were placed in glass bottles and sealed with silica gel septum and thereafter incubated in darkness for 2.5 h at room temperature. An amount of 1 mL gas sample was extracted for the detection and analysis of ethylene by a meteorological chromatograph (GC5890, Cojet, Nanjing, China). It was advisable to maintain the packed injector temperature at about 55 °C, the oven temperature at about 85 °C and the flame ionization detector at about 300 °C. The carrier gas flow rate was fixed at about 30 mL min^−1^ for operation, and the flow rates of air and hydrogen were set at 450 mL min^−1^ and 45 mL min^−1^, respectively. Under these conditions, the retention time for ethylene should be about 0.97 min. Normally, 1.5 mL gas from the headspace of the vials was withdrawn and injected into GC using 1 μL L^−1^ (1 ppm) certified ethylene as the standard. The ethylene peaks were quantified by Turbochrome Navigator software (Perkin-Elmer, Beaconsfifield, Buckinghamshire, UK). Data were from three independent experiments with at least three replicates each.

### 4.12. Statistical Analysis

The whole experiment was performed with at least three biological replicates. The data were analyzed using Microsoft Excel 2016 and SPSS 19.0 software (SPSS Inc., Chicago, IL, USA) for difference analysis. The significance of the mean differences between treatments was analyzed with Duncan multiple comparison at *p* < 0.05.

## 5. Conclusions

CsSAMS1 interacts with the CsCDPK6 protein in cucumber. *CsCDPK6* is utilized in response to a variety of abiotic stresses and hormone responses. Under salt stress, overexpression of the *CsCDPK6* gene in tobacco decreased the stomatal opening and density, and the MDA content, H_2_O_2_ level and electrolyte permeability were lower than those of WT, significantly improving salt tolerance. In addition, repression of the *CsCDPK6* gene increased endogenous ethylene synthesis in cucumber; under salt stress, *CsCDPK6*-silenced lines promoted the transformation from Put to Spd and Spm in cucumber.

## Data Availability

Not applicable.

## Supplementary Materials

The following are available online at https://www.mdpi.com/article/10.3390/ijms222011133/s1.
