# Supplementary material for: CsCDPK6, a CsSAMS1-Interacting Protein, Affects Polyamine/Ethylene Biosynthesis in Cucumber and Enhances Salt Tolerance by Overexpression in Tobacco
Source: Int J Mol Sci. 2021 Oct 15;22(20):11133. doi: 10.3390/ijms222011133 (PMC8538082; doi:10.3390/ijms222011133)
Supplement: Supplementary file 1 [file ijms-22-11133-s001.zip › ijms-1391084-supplementary.pdf]

Table S1: Primer sequences

Primer	Sequence (5′-3′)
CsCDPK6-F	ATCAACCCAGCACAACCGATAC
CsCDPK6-R	CAAGGAATCACCCCGCTTT
pGBKT7-SAMS1-F	GGCCATGGAGGCCATGGAGACCTTTC
pGBKT7-SAMS1-R	CCGCCCGGGAATTAAGATTGAGGCT
pGADT7-CDPK6-F	agattacgctcatatggccatggaggccATGGGTGTTTGTTTTTCGG
pGADT7-CDPK6-R	cagctcgagctcgatggatcccAATATGAGGTCTTGAACTAATAC
SAMS1-NF (SAMS1^{n}-NF)	tcaccatttacgaacgatagttaattaaATGGAGACCTTTCTATTT
SAMS1-NR (SAMS1^{c}-NR)	ccaccactgccacctcctccactagtAGATTGAGGCTTCTCCCAC
CDPK6-CF	tcaccatttacgaacgatagttaattaaATGGGTGTTTGTTTTTCGGC
CDPK6-CR	ccaccactgccacctcctccactagtAATATGAGGTCTTGAACTAAT
SAMS1^{n}-NR	ccaccactgccacctcctccactagtGTGAACACCCTGAGCGATA
SAMS1^{z}-NF	tcaccatttacgaacgatagttaattaaATGGGCCACTTCACAAAG
SAMS1^{z}-NR	ccaccactgccacctcctccactagtGACAAAACGGCCTGAAGGG
SAMS1^{c}-NF	tcaccatttacgaacgatagttaattaaATGATTGGTGGCCCTCATG
pGEX-FLAG-F	CTCAAGCAATCAAGCATTCTAC
pGEX-FLAG-R	AGGACACACTTTAACAATAGGCGA
pCsVMN-HA-F	TACTGAGGATACAACTTCAGAGAAA
pCsVMN-HA-R	GAAGCGTAATCTGGAACATCATAC
CsCDPK6-FLAG-F	cgcggtggcggccgctctagaATGGGTGTTTGTTTTTCGGCC
CsCDPK6-FLAG-R	ttcctgcagcccgggggatccAATATGAGGTCTTGAACTAATACTTGCA
CsSAMS1-HA-F	aattctgcagtcgacggtaccATGGAGACCTTTCTATTTACCTCAGAA
CsSAMS1-HA-R	tgggtaacctgccatggatccAGATTGAGGCTTCTCCCACTCGCAGACGAG GG
Tobacco-CDPK-F	ttacaattaccatggggcgcgccATGGGTGTTTGTTTTTCGGCC
Tobacco-CDPK-R	aacatcgtatgggtaggtaccAATATGAGGTCTTGAACTAATACTTGCA
pV190-F	CGATCAGTCTATTGTCCTAG
pV190-R	ATATCTACGACAGACGAGGG
pV190-CDPK6-F	aggactttacttaatggatccATGGGTGTTTGTTTTTCGGCC
pV190-CDPK6-R	cctagacctataactggatccATACGTATAACCAAATTGACCGTGTC
